# Danish endodontic practice-based research network: follow-up data

**DOI:** 10.2340/aos.v84.43857

**Published:** 2025-06-10

**Authors:** Lise-Lotte Kirkevang, Lotte Hein Sørensen

**Affiliations:** a Department of Dentistry and Oral Health, Aarhus University, Aarhus C, Denmark; b Private Dental Practice, Fredensborg, Denmark

**Keywords:** Practice-based research, endodontics, follow-up, longitudinal, clinical study

## Abstract

**Objective:**

The aim of this prospective, clinical study was to follow-up root canal treatments performed by dentists in the practice-based endodontic research network setting, to assess treatment outcome and factors related to treatment outcome.

**Material and methods:**

Baseline information from 536 teeth was included, follow-up information on 269 teeth (50%) was available. Treatments were performed by 12 dentists during 2017–2018. Follow-up period varied from 3.5 months to 3 years. Treatment outcome was assessed in periapical radiographs using the Periapical Index (PAI). Cases were classified as referred or not. Pre-, intra-, and post-operative variables were recorded by the dentists during treatment and analysed at follow-up in relation to treatment outcome.

**Results:**

Periapical status improved or remained healthy in 174 (66%) teeth. If PAI at baseline was 3, 4, or 5, the outcome was affected negatively (*p* = 0.049). The length and seal of the root filling was assessed adequate in 75% and 63% of the teeth, respectively. Adequate seal (*p* = 0.02) and length (*p* = 0.03) resulted in improved treatment outcome.

**Conclusions:**

When initial periapical status and/or quality of the root filling was good, there was a higher chance of a successful treatment outcome.

## Introduction

Patient-based cohort studies or randomised controlled studies are most often performed in controlled clinical environments such as Dental Schools, Universities, or dental specialist practices. The root canal treatments are performed on highly selected patient groups and teeth, by an endodontist or a dentist with special training in endodontics, and a specific treatment protocol is followed strictly. The overall success rate for root canal treatments in clinical outcome studies performed up till 2002 was estimated to be approximately 74.7%, and in studies performed from 2003 to 2020 the success was estimated to be slightly higher, 82.0% [1, 2].

Translation of knowledge from one environment to another has proven to be difficult, which in turn may delay implementation of new knowledge from research in general practice [3, 4]. To overcome the gap between the research environment and the general clinic, establishment of practice-based research networks has been proposed [3–5].

Most recently, the European Society of Endodontology developed a comprehensive clinical guideline for diagnosing and treating pulpitis and apical periodontitis [6]. This guideline aimed at describing the effectiveness of different treatments and treatment procedures. Effectiveness is the ability of a treatment intervention to have a meaningful effect on patients in normal clinical conditions. However, since most clinical outcome studies have been performed in highly controlled environments, it was evident that there is a lack of studies including clinical information on treatment procedures and treatment outcomes performed in general dental practice.

In Denmark, there is no recognised specialty in endo-dontics. However, a number of dentists are dedicated to endodontics, and they work almost exclusively with referred cases. They get more experience and become more competent in treating endodontic cases, and thus receive more referrals of complex cases.

To investigate clinical factors related to endodontic treatment outcome in general dental practice, there is a need for studies which systematically record the treatment procedures and follow the results over time. In Denmark, baseline data from a practice-based endodontic research network reported that the participating dentists overall followed internationally recom-mended guidelines regarding endodontic treatments [5, 6]. It was shown that among the participating dentists, newer technology such as mechanical instrumentation and use of magnification was high, that rubber dam was frequently used, and that sodium hypochlorite (NaOCl) was preferred for irrigation [5].

The purpose of the present study was to follow-up root canal treatments performed by dentists in the practice-based endodontic research network in Denmark, to assess treatment outcome and to identify factors related to treatment outcome in this setting.

## Materials and methods

The project was described and sent to the Science Ethics Committee region Midtjylland (notification number: 57871) and the Danish Data Protection Agency. The project was financially supported by the Danish Endodontic Society.

**Figure 1:**
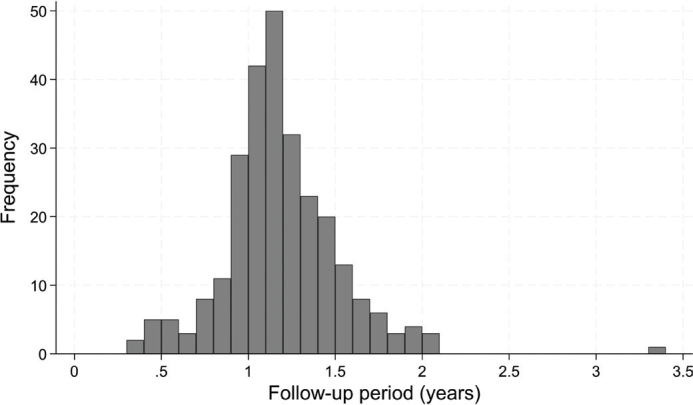
Frequency distribution of length of follow-up period.

The baseline data were collected during the period November 2017 to July 2018. Patients gave written consent to participate and consented to be scheduled for a control visit after one year. The follow-up period ranged from 3.5 months to 3 years (Figure 1).

Of 581 teeth registered at baseline [5], 45 teeth were surgical endodontic cases and not included in the present study. Eventually, follow-up data for 269 of the 536 cases were retrieved (50%). Four dentists (*n* = 22 teeth) were not able to participate in the follow-up investigation for personal reasons. Four teeth had been extracted prior to follow-up. No specific reasons for the lack of follow-up information were available for the remaining non-participating teeth.

### Registrations

Registrations at baseline included age, gender, and treatment date, together with clinical and radiographic information. Cases were classified as referred or not. The clinical factors were registered by the treating dentist before, during, and after the treatment. A detailed description was provided in a previous study [5]. At follow-up examination, the dentist registered the date of the follow-up examination and if the tooth was still present. The periapical status was assessed using the Periapical Index (PAI) at baseline and follow-up [7].

The same two observers who assessed the baseline data also assessed the follow-up radiographs. Before the assessment, the observers underwent a calibration course for the PAI system [7]. The observers’ scores were compared to a reference standard, resulting in a Cohen’s Kappa score of 0.69 and 0.81, respectively. The radiographs were assessed individually; in case of disagreement the case was discussed until consensus was reached. The consensus scores were used for all data treatment and analyses. The prognostic factors considered in the present study are described in Table 1.

**Table 1:** Prognostic factors recorded at baseline. Periapical status was assessed at follow-up. (number and percentages).

Prognostic factors	Categories	Number of teeth (%)
Referral dentist	Yes	126 (47)
	No	143 (53)
Tooth group	Front	36 (13)
	Premolar	41 (15)
	Molar	192 (71)
Periapical Index (PAI)	1	64 (24)
	2	9 (3)
	3	118 (44)
	4	51 (19)
	5	27 (10)
Preoperative pain (VAS)†	No (0–4)	204 (24)
	Yes (5–10)	65 (76)
Swelling	No	249 (93)
	Yes	20 (7)
Primary treatment	Root canal treatment, vital pulp	52 (19)
	Root canal treatment, necrotic/infected pulp	158 (59)
	Non-surgical retreatment	59 (22)
Aseptic measures^a^	Rubber dam	231 (87)
	Cotton rolls, suction	35 (13)
Root filling quality, seal^b^	Adequate	168 (63)
	Inadequate	100 (37)
Root filling quality, length^a^	Adequate	201 (76)
	Long	28 (11)
	Short	37 (14)
Patency^a^	Yes	138 (52)
	No	128 (48)
Sealer^b^	Surplus	148 (55)
	No surplus	120 (38)
Coronal restoration, quality^c^	Adequate	199 (75)
	Inadequate	65 (25)
**Follow-up**		
Periapical Index (PAI)^d^	1	129 (49)
	2	23 (9)
	3	62 (24)
	4	31 (11)
	5	17 (6)

Missing values:

a 3,

b 1,

c 5,

d 7.

† Visual Analog Scale.

### Data treatment

Registration forms and radiographs were sent to the Department of Dentistry and Oral Health, Aarhus University, where they were recorded, systematised, and coded. All variables, except age and follow-up time were categorical.

The proportion of referred cases was used to assess the experience of the dentists. If > 80% of the treated cases from a dentist were referred treatments, the dentist was characterised as an endodontic referral dentist. Chi-square test was performed to describe if treatments differed between referral and not referral dentists.

The quality of the root canal treatment was assessed in relation to length and seal on the baseline radiographs. The length was assessed adequate if the root filling was assessed to end < 2 mm from radiographic apex, short if it was assessed to end > 2 mm from radiographic apex, and long if the gutta-percha extended beyond the radiographic apex. Sealer surplus was assessed separately. The seal was assessed adequate if no voids were seen in the apical half of the root filling (Table 1). The coronal restoration was assessed adequate in no open margins or material surplus was detected on the radiographs.

In the present study, the outcome was derived from changes in PAI score from baseline to follow-up examination. Extraction was included as a sixth and worst category. Baseline PAI scores were cross tabulated with follow-up PAI scores and information on extraction. A signed rank test was performed to assess changes (Table 2).

**Table 2:** Follow-up periapical index (PAI) scores versus baseline PAI scores, including tooth extraction at follow-up as a separate category (number and percentages).

Baseline PAI scores	Follow-up PAI scores					Tooth extraction	Total (%)
	1	2	3	4	5
1	46	6	5	3	2	0	62 (23)
2	8	0	1	0	0	0	9 (3)
3	54	13	33	10	5	4	119 (45)
4	15	1	16	13	5	0	50 (19)
5	6	3	8	4	5	0	26 10)
Total (%)	129 (48)	23 (9)	63 (24)	30 (11)	17 (6)	4 (2)	266 (100)

The size of the study did not allow for a comprehensive analysis of the relative importance of the different factors. However to assess prognostic factors, crude and adjusted logistic regression analyses were performed. For these analyses, the outcome was dichotomised: if follow-up PAI was lower than baseline PAI or if baseline PAI continued to be 1 or 2, the outcome was considered successful. Consequently, if follow-up PAI was higher than baseline PAI or if baseline PAI continued to be 3, 4, or 5, or if the tooth had been extracted, the outcome was considered unsuccessful. In the adjusted analyses, the effects of primary treatment, tooth group, and referral dentist were corrected for. Consequently, a prognostic factor was assessed by comparison of cases that received the same primary treatment, were from the same tooth group, and were treated by dentists with comparable levels of experience. Odds ratios (OR) > 1 indicated increased chance of success (Table 3). Analyses of non-participation was performed using Chi-square test (Table 4). Statistical software Stata (StataCorp, College Station, TX, USA; release 18) was used for data management and statistical calculations.

**Table 3:** Prognostic factors for a successful outcome. Crude and adjusted logistic regression analyses, including odds ratios (OR) and 95% confidence intervals (CI).

Variable	Crude OR	*p*	Adjusted OR*	95% CI	*p*
**Tooth group**		0.39			0.39
Front	1		1	ref.	
Premolar	1.95		1.96	0.75; 5.12	
Molar	1.37		1.35	0.85; 2.84	
**Primary treatment**		0.70			0.71
Root canal treatment, vital pulp	1		1	ref.	
Root canal treatment, necrotic/infected pulp	0.75		0.74	0.36; 1.53	
Non-surgical retreatment	0.81		0.80	0.33; 1.93	
**Referral dentist**		0.74			0.96
No	1		1	ref.	
Yes	0.95		1.01	0.58; 1.78	
**Apical periodontitis**		0.04			0.049
PAI scores 1, 2	1		1	ref.	
PAI scores 3, 4, 5	0.52		0.50	0.25; 1.00	
**Preoperative pain**		0.15			0.19
Yes (5–10)	1		1.00	ref.	
No (0–4)	0.63		0.62	0.30; 1.27	
**Swelling**		0.29			0.39
Yes	1		1	ref.	1
No	1.65		1.51	0.59; 3.88	
**Aseptic measures**		0.88			0.72
Rubber dam	1		1	ref.	
Cotton rolls, suction	0.94		0.86	0.38; 1.94	
**Root filling quality, seal**		0.02			0.02
Adequate	1		1	ref.	
Inadequate	0.52		0.53	0.31; 0.90	
**Root filling quality, length**		0.03			0.03
Adequate	1		1	ref.	
Inadequate (long/short)	0.53		0.50	0.28; 0.95	
**Patency**		0.29			0.24
Yes	1		1	ref.	
No	0.76		0.73	0.43; 1.23	
**Sealer**		0.81			0.82
Surplus	1		1	ref.	
No surplus	1.07		1.06	0.61; 1.88	
**Coronal restoration, quality**		0.17			0.17
Adequate	1		1	ref.	
Inadequate	0.67		0.66	0.37; 1.20	

* Adjusted analyses: Primary treatment, tooth group and referral dentist are mutually adjusted. The remaining factors are adjusted for these three variables.

**Table 4:** Analyses of teeth available at follow-up (participation) and teeth not available at follow-up (non-participation). Number of teeth and p-values, analysed using Chi-square test.

	Non-participation	Participation	*p*
**Primary treatment**			
Root canal treatment, vital pulp	66	52	
Root canal treatment, necrotic/infected pulp	143	158	
Non-surgical retreatment	55	59	0.29
**Tooth group**			
Front	29	36	
Premolar	70	41	
Molar	165	192	0.01
**Referral dentist**			
No	154	143	
Yes	110	126	0.23

## Results

There were 148 women (55.0%) and 121 men (45%) among the included follow-up patients. The age varied between 10 and 90 years, with a mean of 56.1 years. The mean number of root canal treatments performed by each dentist was 22.6, with considerable variation from 3 to 72 treatments.

The teeth with follow-up information did not differ much from the teeth where no follow-up information was available, except for fewer premolars being available for follow-up (Table 4). Four molars had been extracted at follow-up (after 1.2, 1.4, 1.6, and 2.0 years). All the extracted teeth had PAI score 3 at baseline.

Almost 60% of the root canal treatments were teeth with necrotic/infected pulps, and approximately 20% were either retreatments or teeth with a vital pulp. The number and distribution of the prognostic factors are shown in Table 1. Molars were by far the most often treated tooth (71%) followed by premolars (15%) and front teeth (13%).

Referral dentist treated more molars, and more teeth with non-vital pulps, with or without previously performed root canal treatment. They consistently used rubber dams, the patency technique, and had sealer surplus.

**Figure 2:**
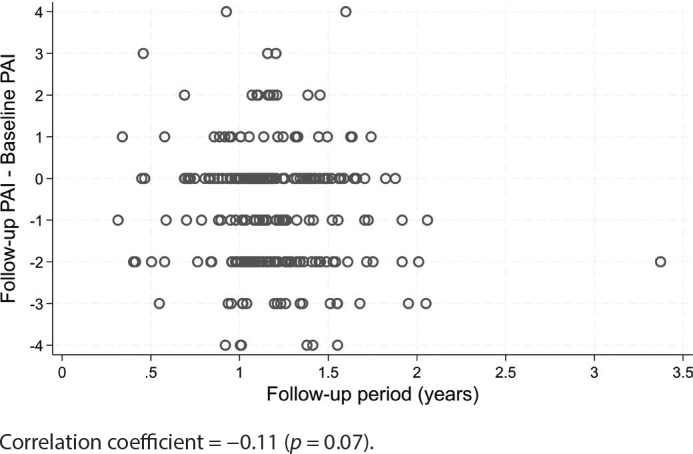
Change in PAI score according to length of follow-up period.

In Table 2, the baseline PAI scores were cross tabulated with follow-up PAI scores and information on extraction. At follow-up, the periapical status had improved in 174 teeth (66%) (*p* < 0.001). There was a tendency for improvement in PAI score with longer follow-up periods (*p* = 0.07) (Figure 2).

In the final, adjusted analyses, teeth without apical periodontitis at baseline (PAI score 1 or 2) had a significantly improved chance of a successful outcome compared to teeth with apical periodontitis at baseline (PAI scores 3, 4 or 5) (Table 3).

The quality of the root canal treatment affected the outcome. When the root filling seal was assessed adequate, a significantly increased chance of a successful outcome was seen, and likewise the chance of a successful outcome was increased if the root filling length was assessed adequate. Regarding root filling length, however, an impaired outcome was related to short rather than long root fillings. Several factors did not show an effect on treatment outcome; the use of patency technique, sealer surplus, the quality of the coronal restoration at baseline, preoperative pain, and the use of rubber dam (Table 3).

## Discussion

Of the 536 teeth which were analysed in the previous baseline article, 269 root filled teeth (50%) were available for follow-up. Non-participation is a well-known problem in clinical research and impossible to avoid. Therefore, a non-participation analysis on tooth level was performed and this analysis did not reveal any serious differences between teeth that participated in the follow-up, and teeth that only participated in the baseline investigation. In a recent review of clinical outcome studies performed from 2003 to 2020, participation rates of 23–100% were reported [2].

The follow-up period ranged from 3.5 months to 3 years, with most of the treatments being followed from 1 to 1.5 years (Figure 1). The dentists had been asked to do a 1-year follow examination. However, for a part of the patients this coincided with the COVID period, and the restriction related to that. In some cases, the dentist may have decided to use information from a previous visit and in some cases the recall may have been postponed. In clinical studies collaboration of patients is crucial, but they do not always respond as we would prefer, in particular in relation to recalls.

The periapical status was assessed using the PAI. The development of the PAI was based on a previous study from 1967, comparing histological and radiographic appearances of periapical changes in human autopsy material from 292 maxillary incisors (142 individuals) [8]. The study concluded that that a continuous inflammatory process could be detected radiographically, and in 1986 this formed the basis for development of the PAI [7]. It may be discussed if the PAI system is valid for canines, premolars, and molars since the histologic material, on which it was based, only included maxillary incisors. Anatomic structures vary and could affect the diagnostic process, but this problem would apply to all radiographic classifications of apical periodontitis. During the last decades, 3D radiography has emerged. However, to date no other radiographic classification for diagnosing apical periodontitis, based on either 2D or 3D and verified by histology, has been proposed.

The periapical status was assessed at baseline and follow-up, and in the present study the full scale of PAI was used to detect improvement or impairment of the periapical health. Thus, the changes in PAI score from baseline to follow-up examination were considered, and extraction was included as a sixth and worst category. This approach was previously used in an observational study of the general Danish population with focus on changes in periapical status. In that study, it was shown that each score in PAI had its own prognostic value when a tooth was assessed after an observation period of 5 years [9].

In the present study, the observation period was considerably shorter; but it was shown that baseline PAI had a significant effect on the treatment outcome with lower baseline PAI scores having highest chance for a successful outcome after follow-up. It was also seen that longer follow-up period affected the treatment outcome positively. Follow-up time may play an important role in the estimation of treatment outcome and should be accounted for when reporting treatment results [10]. There has been considerable variation in outcome reporting among clinical studies on non-surgical root canal treatment [11], and an attempt to develop a core outcome set for endodontic treatments has been initiated [12, 13]. In the recent development of clinical guidelines for endodontic treatment, a minimum follow-up time of 1 year was suggested for assessment of periapical health after root canal treatment [6].

In the present study, 25% of the patients experienced preoperative pain ranging from 5 to 10 on a scale of 0–10, where ‘0’ corresponds to no pain/discomfort and ‘10’ to unbearable pain/discomfort. The preoperative pain reduced considerably 1 week post-operatively [5]. At follow-up preoperative pain did not show an effect on treatment outcome (Table 3). Preoperative pain was also not reported to affect treatment outcome in a United States of America practice-based research study [14]. In a systematic review on persistent tooth pain, it was shown that persistent pain after root canal therapy was uncommon [15].

As expected, a higher proportion of retreatments and treatments of molars was found among the teeth treated by the referral dentists. There was also a difference in treatment procedures, but the root filling quality did not seem to differ between the referral dentists and the other dentists, and treatment outcome was not significantly different (Table 3). This was in accordance with a recent review that found no difference in treatment outcome among undergraduate students, general dentists, post-graduate students, and endodontic specialists [2].

It is evident that specialists perform more complicated treatments such as molars, teeth with obliterated root canals, retreatments, and surgical endodontic cases [14, 16–18]. However, even in countries, which have specialist education programmes in endodontics, most endodontic treatments are performed by general dentists [14, 17, 18]. As specialists perform more difficult treatments where the prognosis for the treatment is poorer compared to less complicated treatments, it is difficult to compare treatment results directly. Studies have shown that treatment outcome does not seem to differ much between specialists and generalists, even though the specialists perform more difficult treatments [14, 19, 20]. However, an improved 10-year survival rate of molars treated by endodontists compared to non-endodontists has been reported [21]. The use of rubber dam was high in the present study, in particular among the referral dentists [5]. Surprisingly, however, the use of rubber dam did not significantly affect the chance of a successful outcome; although, a slight tendency for treatments performed without rubber dam to have a lower chance of a successful outcome was seen (Table 3).

Overall, the quality of the root canal treatment assessed in radiographs was relatively high in the present study, with 63% and 75% assessed adequate for seal and length, respectively (Table 1). In the review by Ng et al. (2008), only few of the included studies included information on root filling quality, but in these studies the overall proportion of adequate root fillings was 84% [1]. Unfortunately, a more recent review of outcome studies did not report on root filling quality [2]. In the present study, the quality of the root canal treatment significantly affected the treatment outcome. Teeth with a root filling assessed to have an adequate length and an adequate seal, had a higher chance of an improved periapical health (Table 3). The importance of a good quality root filling was also documented in reviews on both primary and secondary root canal treatments [1, 22]. Most often, the root filling quality has been used as a surrogate measure for the quality of the entire root canal treatment, and it is reassuring that the data from the present study support the value of this parameter when assessing the treatment outcome. In fact, the data from the present study indicate that the root filling quality parameters may possess a higher prognostic value than specific clinical parameters (Table 3).

The coronal restoration quality at baseline was assessed adequate in 76% of the treated teeth; there was a tendency for inadequate coronal restorations to be associated with a poorer outcome compared to the adequate restorations, but the effect was not statistically significant perhaps due to the limited number of cases (Table 1). Both observational and clinical outcome studies have previously reported an association between a good quality coronal restoration and periapical health, both in observational studies [1, 2, 23, 24].

## Conclusion

Overall, an improved or continued healthy periapical status was seen in 66% of the root canal treated teeth. When the initial periapical status and/or the quality of the root filling was good, there was a higher chance of a successful treatment outcome.
